# Life review for older adults: an integrative review

**DOI:** 10.1111/psyg.13194

**Published:** 2024-10-08

**Authors:** Vincent Jiang, Alexandra Galin, Xanthe Lea

**Affiliations:** ^1^ Sydney Medical School, University of Sydney Sydney New South Wales Australia; ^2^ Central Clinical School, Royal Prince Alfred Hospital Sydney New South Wales Australia; ^3^ Faculty of Medicine and Health Sydney Nursing School, University of Sydney Sydney New South Wales Australia

**Keywords:** elderly, integrative review, life review, life review therapy, older adults

## Abstract

Life review therapy is a form of psychotherapy framework which involves the guided reflection of life events throughout a patient's life journey. Patients are encouraged to actively recall and analyze important life events, both negative and positive. Through this process, patients may be able to come to terms with, or even resolve negative events such as conflicts and regrets. The aim of this study is to gain an understanding of the current knowledge of the use of life review therapy in older adults, and to identify areas for future research. A systematic literature search was conducted across CINAHL, Medline, PsycInfo, Embase and Scopus. Papers were screened and selected using predetermined inclusion and exclusion criteria using Covidence and exported into Excel. Data analysis was conducted to synthesise thematic analyses. One hundred and thirty‐one articles from 1974 to 2023 were included in this study and were used to develop four themes: life satisfaction and self‐esteem, depression and depressive symptoms, institutional care and cognitive decline, and post‐traumatic stress disorder (PTSD) and trauma. Life review has some benefit in older adults in certain groups and situations, although the extent and duration of effectiveness of this benefit is unclear. Life review unlikely improves self‐esteem. However, in the short term, life review appears to improve life satisfaction, depression or depressive symptoms, and PTSD symptoms in the elderly. Life review may be a useful therapeutic tool for older adults with cognitive decline for as long as the intervention continues. Areas for future research are explored.

## INTRODUCTION

The ageing population continues to grow, with the number of people in the world over 65 years of age expected to more than double, from 721 million in 2021 to possibly 1.6 billion in 2050.^1^ Depression, anxiety and social isolation are commonly associated with the process of ageing,^2^ with some studies estimating the prevalence of depression within this population to be 32%.^3^ Common consequences of ageing such as decreased physical health, increased life stressors, and psychosocial factors such as decreased social network, are associated with depression within older adults.^4^ Some studies have shown the correlation of repetitive recollection of regrets with decreased mental well‐being and general distress.^5^

### The life review process

In Butler's seminal paper on life review^6^ the process of life review and reminiscence was described as a common and normal process in ageing. In particular, he observed this phenomenon to be particularly common in those in closer proximity to death, of which the aged constitutes a large proportion.^6^ This process can be quite cathartic and therapeutic for many older adults, and through successful integration and reflection of life events it allows older adults to achieve ego integrity, as described in Erikson's stages of psychosocial development.^7^ However, where there are many unresolved regrets and conflicts, the intrinsic process of life review can result in the development of depression, anxiety, despair, and other mental health comorbidities. The resultant pathological forms of reminiscence such as rumination or obsession are associated with unsuccessful ageing with poorer outcomes.^8^ Therefore, facilitated and structured life review therapy is thought to help identify, disentangle and reframe these problematic and unreconciled thought patterns or behaviours.^9^

### Life review as an intervention

Life review therapy is a form of psychotherapy framework originally described by Butler^10^ which involves the guided reflection of life events throughout a patient's life journey. Based on existing cognitive processes associated with ageing such as reminiscence,^6^ this technique has been revised over the years, such as with the added use of memorabilia (photos or sentimental possessions) to stimulate memories.^11^ Many practitioners now follow Haight's systematic and replicable methodology for individual therapy where there is chronological structure with a distinct beginning (childhood) and end.^9^ Further research has noted the benefit of longer sessions and wider scope (encompassing the entire life cycle),^12^ which has influenced how life review is conducted in modern times. Since then, structured life review has been adapted into numerous formats such as individual, dyadic^13^ or group forms.^14^ While traditionally conducted in oral form, life review has also been adapted into different modalities such as written forms,^15^ computer supplemented^16^ or music‐based forms^17^ just to name a few. Despite these differences, in all forms of structured life review, participants are encouraged to actively recall, analyze and integrate important life events throughout the life span, both negative and positive. Through this process, participants may be able to come to terms with, or resolve negative memories such as conflicts or regrets which may have previously harmed their sense of self‐worth and life satisfaction. Contemporaneously, participants are able to revisit and re‐emphasise overlooked positive memories from which they can derive joy and contentment, and ultimately reframe their perspectives on their lives in more positive light.^18^ In this paper, life review interventions will be defined as such, where the content is consistent with these concepts.

## AIMS

The aim of this study is to gain an understanding of the current state of knowledge about the use of life review therapy in older adults, and to identify areas where future research may be indicated.

## METHODS

This study was conducted using the Whittemore and Knafl's integrative review study design.^19^ This involves the collection of a broad range of literature including empirical and non‐empirical research, quantitative and qualitative. Given the abundance of literature and high variability of study designs for life review interventions, this method allowed the authors to conduct a broad search and gain a through overview of the current existing literature which subsequently could be integrated and utilised to identify areas for future research. Following this methodology, a systematic literature search was conducted, and data extracted, analyzed and integrated to generate thematic conclusions.

### Search strategy

**Figure 1:**
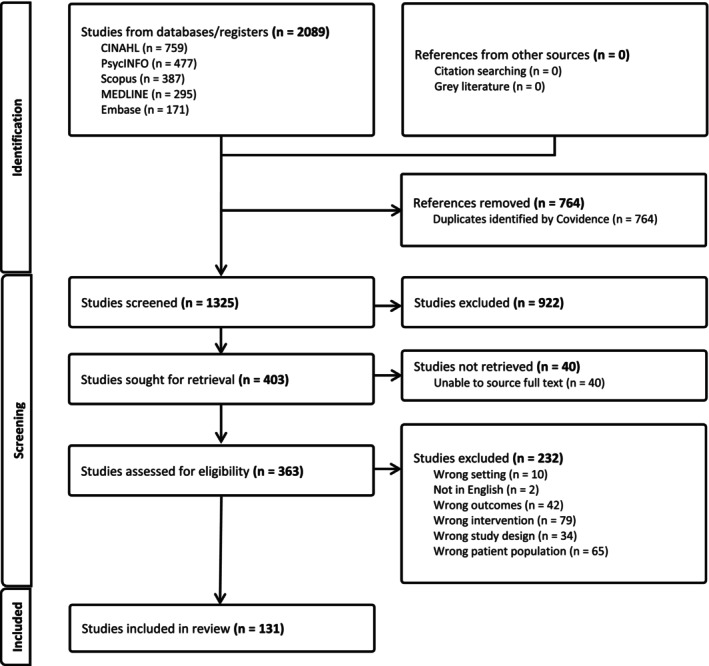
Preferred Reporting Items for Systematic reviews and Meta‐Analyses (PRISMA) flow diagram of included studies.

A systematic search across multiple electronic databases including CINAHL, Medline, PsycInfo, Embase and Scopus was conducted by the authors on June 8, 2023. No date limit was utilised and resulted in 1325 articles from the years 1974 to 2023 after duplicates were removed via Endnote and Covidence. A date limit was not included to ensure as comprehensive search as possible, especially given a large number of important studies were conducted in the 20th century. The final search strategy keywords included: (life review OR life review therapy OR life history review) AND (older adult OR older person OR elderly OR geriatric patients OR aged OR over 65). More detail about the search strategy can be found in Fig. 1 and Appendix A.

### Inclusion and exclusion criteria

Inclusion criteria required papers to have: (i) life review in any form as the intervention; (ii) study participants of age 60 years or above; (iii) qualitative, quantitative, or mixed methodologies, review articles and textbooks; (iv) published any time; and (v) available in English. Where age was not explicitly stated, studies were still included if the population was defined as older adults and related synonyms. Exclusion criteria were: (i) study participants aged less than 60; (ii) editorials, commentaries; and (iii) not available in English. It is important to note, the original inclusion criteria set the age criteria to 65 and older. However, during the full text screening stage, a large proportion of papers defined older adults as age 60 and older, which we subsequently decided was an appropriate age gap. This was to ensure that older research with potentially lower cutoffs for the definition of elderly (conducted when lifespans were comparatively shorter) would not be excluded from the literature search.

### Screening and data extraction

Our research team comprised of two primary researchers (VJ and AG) and one supervising researcher (XL). Screening and data extraction were conducted on Covidence by both primary researchers. The Covidence workflow for this study comprised of a title and abstract screen, full text screen and data extraction stage. Papers in the title and abstract screen were screened once by either primary researcher, and papers in the full text screen were screened by both primary researchers. Where there were disagreements for whether papers should be included, supervising researcher (XL) was invited to contribute to the decision. Data were extracted using the Covidence data extraction tool, and the researchers extracted data according to a modified Covidence data extraction template. The following data were extracted: title, authors, aim of study, delivery modality of therapy (individual, group, mixed therapies), study design, conflicts of interest, whether intervention was beneficial to the population group (as stated in authors' conclusions and statistically significantly improved outcome scores where available), sustained benefit, themes, conclusions and results/salient findings. Themes were identified by both authors during the full text screening stages and each study was tagged according to which themes it was related to. During the data extraction stage, more themes were identified and were added into the existing tags. These data were subsequently exported into Excel, upon which thematic analysis was conducted. The full list of literature included for the generation of the results of this research is available in Appendix B.

### Data analysis

The initial search produced 2089 papers, and after duplicates were removed, resulted in 1325 papers for title/abstract screening. After full text screening, a total of 131 articles meeting inclusion and exclusion criteria were included in the study (Appendix B). A flowchart demonstrating this process can be seen in Fig. 1. These studies were grouped by study design, including 11 systematic reviews, 16 randomised controlled trials (RCTs), 54 non‐RCT experimental studies, 20 case reports/case series. Non‐RCT experimental studies included all experimental cohort studies that were not randomised controlled trials. These were grouped together because the experimental studies were highly variable in study design and methodology. Non‐empirical research, of which there were 30 papers, comprised of non‐empirical research papers including non‐systematic literature reviews, guides, textbooks, and thematic analyses. Papers included in this study were published between 1974 to 2023.

All forms of evidence were included in the development of themes. While no study was excluded on the basis of quality, greater weight was attributed to higher levels of evidence with more rigorous study design such as systematic reviews and RCTs. Themes were generated from the table of extracted data based on patterns and abundance of research pertaining to each theme, in accordance with the Whittemore and Knafl integrative review method.^19^

## RESULTS

### Theme 1: Life satisfaction and self‐esteem

Fifty‐seven articles investigated life review in the context of life satisfaction. Life satisfaction was measured using a number of questionnaires and variants of life satisfaction indexes such as Life Satisfaction Index A (LSIA).^20^ Of these included studies, the empirical evidence comprised of six systematic reviews, 10 RCTs, 20 non‐RCT experimental studies, six case reports and two cohort studies. The remainder comprised of qualitative studies and theoretical discussions. Three of the systematic reviews concluded that life review was effective in improving life satisfaction,^21^, ^22^, ^23^ two had potential but unconfirmed benefit (due to insufficient evidence)^24^, ^25^ and one study did not find benefit.^26^ Seven of the RCTs concluded improvement in life satisfaction^17^, ^27^, ^28^, ^29^, ^30^, ^31^, ^32^ with the remainder with no improvement.^16^, ^33^, ^34^ However, some of these studies noted the integrated benefit of socialisation which helped to meet unmet social needs of older adults.^34^ Two RCTs investigated sustained benefit, ranging from 1 month^29^ to 3 months.^32^ Based on these data, the larger majority of the current literature suggests life review appears to improve life satisfaction in the short‐term and may have some sustained benefit. The theory would suggest this is due to the recall of positive memories and reconciliation of negative ones.^35^ With this integration of memories and life events, individuals can have readapted perspectives on their lives and potentially achieve gerotranscendence^36^ or ego integrity. It may also be through the rediscovery of the meaning of life for the individual.^37^ Given this, more research is required to clarify or affirm the long‐term benefits on life satisfaction from life review.

Self‐esteem is a commonly investigated aspect of the healthy ageing process, often measured using self‐esteem scales such as the Rosenberg Self‐Esteem Scale (RSES).^38^ Eighteen articles explored the benefits of life review for self‐esteem. Results from the two systematic reviews measuring self‐esteem determined no improvement^22^ or potential but uncertain improvement.^25^ While significant improvements in self‐esteem were found in both RCT studies,^16^, ^29^ only three non‐RCT studies found improvement in self‐esteem scores after therapy,^39^, ^40^, ^41^ with the six remaining non‐RCT experimental studies demonstrating no improvement^42^, ^43^, ^44^, ^45^, ^46^ or potential but unclear improvement.^47^ It is important to note that of the non‐RCT studies that demonstrated benefit, the intervention comprised of hybrid life review forms (combined with cognitive‐behavioural techniques) or patient groups investigated were cognitively impaired, which confounds improvements of self‐esteem due to life review alone. Although three case studies^48^, ^49^, ^50^ and one textbook^51^ suggest that life review can improve self‐esteem, the larger majority of the empirical evidence suggests that with life review this is less often the case.

### Theme 2: Depression and depressive symptoms

Depressive symptoms were the most common outcome investigated in the literature. A total of 73 articles were found whereby life review was used to treat patients exhibiting depressive symptoms or with diagnosed depression. Empirical evidence within this group included 10 systematic reviews, 12 RCTs, 24 non‐RCT experimental studies and eight case reports. Of the systematic reviews, nine studies concluded that life review improves depression symptoms.^22^, ^24^, ^25^, ^52^, ^53^, ^54^, ^55^, ^56^, ^57^ In particular, one systematic review yielded statistically significant improvements in depression scores in meta‐analysis.^55^ The remaining systematic review was inconclusive about benefits for depression due to insufficient number of high‐quality studies.^21^ One systematic review determined that improvements in depressive symptoms may last from 2 weeks up to 3 months,^52^ while another study determined small follow up effects.^53^ All RCTs found significant improvements in depression scores after life review interventions.^15^, ^16^, ^27^, ^28^, ^33^, ^58^, ^59^, ^60^, ^61^, ^62^ In one paper, there were improvements in depression score, but also no difference between intervention and control groups.^63^ However, greater gain was noted when more specific memories were invoked during the process.^63^

Of the 24 non‐RCT experimental studies, 17 studies found improvement in depression scores.^39^, ^40^, ^45^, ^47^, ^64^, ^65^, ^66^, ^67^, ^68^, ^69^, ^70^, ^71^, ^72^, ^73^, ^74^, ^75^, ^76^ The remaining studies did not find improvements in depression scores,^26^, ^42^, ^43^, ^46^, ^77^, ^78^, ^79^ although one study found the process may be enjoyable even if not therapeutic for treating depression.^80^ There may be greater benefits for life review conducted in dyadic forms compared to group formats.^40^ The theory suggests this may be due to re‐motivation through the rediscovery of past strengths, increased socialisation or a resolution of participant's fear of death.^37^ The large majority of high‐level evidence suggest that life review likely improves depressive symptoms in older adults in the short‐term.

### Theme 3: Institutional care and cognitive decline

A significant patient group investigated for life review comprised of older adults in institutional care. Patient subgroups within this category were comprised of chronic illness, palliative care, cognitive decline (such as dementia) and frail elderly in nursing homes.

Forty‐five papers investigated life review in the context of older patients in institutionalised care in some form, most commonly nursing homes. Empirical evidence comprised of three systematic reviews, eight RCTs, 21 non‐RCT experimental studies and seven case studies. Two systematic reviews were focused exclusively on older patients in long‐term care settings, and found that life review improved quality of life^21^ and depressive symptoms^25^ in these patient groups. Bohlmeijer's systematic reviews found potential benefit from individual and group life review interventions;^23^, ^55^ however, these findings were not exclusive for nursing home environments, but also included studies from independent community‐dwelling patients. Interestingly, one systematic review found significantly greater outcomes from life review for older adults living in the community than in institutionalised care.^23^ All RCTs investigating life review interventions in nursing home environments found some form of therapeutic benefit^15^, ^17^, ^30^, ^31^, ^34^, ^61^, ^62^ for older adults. Notably, significant improvements were found in depression scores^15^, ^61^, ^62^ and life satisfaction scores.^17^, ^30^, ^31^ One RCT investigated life review interventions in homebound elderly, and found improvements in life satisfaction, but not depression scores.^27^ Based on these findings, life review has potential utility in improving depression and life satisfaction in institutionalised elderly.

Twelve studies investigated the use of life review in patients with cognitive decline. There were no systematic reviews that researched life review interventions in this patient group. One RCT integrated life review to create life story books for patients with dementia, and found immediate improvements in quality of life after receiving their life story books.^31^ However, these improvements were not sustained after 6 weeks. Of the non‐RCT experimental studies, five studies found some benefit in dementia patients, citing some improvement in disorientation,^44^ depression,^65^, ^70^, ^76^ socialisation.^81^ However, there was no improvement in life satisfaction or self‐esteem,^44^ and follow up was not conducted in any of these studies. Some of the theories would postulate that life review may increase memory stimulation through the recollective processes.^37^ There may also be additional benefits for this group from increased socialisation.^37^ Based on these data, life review interventions for dementia patients have therapeutic benefit; however, benefits gained from life review are unlikely to be sustained thereafter. Instead, life review may have utility for persons with dementia over prolonged periods, but only for as long as the intervention continues.

### Theme 4: PTSD and trauma

Thirteen articles investigated the use of life review interventions in patients with PTSD or a history of traumatic events. There were no systematic reviews within this area. There were two RCTs in this area, which found life review interventions to be both beneficial and likely sustainable.^29^, ^60^ In one of these most recent RCTs conducted on Holocaust survivors, life review interventions did not improve PTSD symptoms immediately post‐treatment; however, at 6 months follow up statistically significant improvements in PTSD symptoms were found.^60^ The other RCT demonstrated statistically significant improvements in life satisfaction and self‐esteem post‐treatment and at 1 month follow up within a Taiwanese veteran patient group.^29^ All of two non‐RCT experimental studies conducted with separate veteran populations also found improvements in depression symptoms, self‐assessed wisdom and life satisfaction.^64^, ^82^ The remaining empirical studies which were case studies found that life review improved life satisfaction, depression symptoms or self‐esteem in multiple patient groups, including Holocaust survivors,^83^ elderly survivors of childhood sexual abuse,^84^, ^85^ lesbian and gay veterans,^86^ World War II veterans^87^ and non‐specified PTSD patients.^88^ These studies demonstrate potentially highly efficacious applications of life review within this specific patient group. Life review is thought to assist patients review the past and reframe their perception of patients' lives by stimulating new interpretations and encouraging successful reintegration of life events.^29^, ^60^ It is also thought to provide a cathartic stimulus and personal outlet that can encourage remembrance and mourning.^89^ Some non‐empirical literature does discuss the risk of distressing participants through recollection, and that practitioners may need to be trained to help mediate complications and facilitate integration.^89^, ^90^ Overall, this area remains under‐researched, and more research would be valuable to affirm these current findings and consolidate mechanisms for how life review can benefit PTSD patients.

## DISCUSSION

The objective of this integrative review was to gain an understanding of the research on life review interventions for older adults 60 years and older. Results from thematic analysis enhanced our understanding of the current knowledge of life review and evaluated population groups and areas where life review was effective or ineffective. Importantly, it was able to identify areas where further research would be beneficial. Four themes were identified in this study: life satisfaction and self‐esteem, depression and depressive symptoms, institutional care and cognitive decline, and PTSD and trauma. It was demonstrated that life review was effective in improving life satisfaction, depressive symptoms, and PTSD in older adults. Life review also benefited institutionalised elderly in the short term with improvements in depression and life satisfaction scores. It was unclear whether these improvements in depression, life satisfaction or other benefits for institutionalised elderly are sustained. Life review may have utility as a therapeutic tool for persons with cognitive decline for as long as the intervention is implemented. Life review may even be a useful non‐pharmacological intervention in delirium prevention. Benefits from life review in PTSD patients are promising and have potential to be sustained, although more research is required to validate this and establish how life review accomplishes this. Based on the current evidence, life review is unlikely to be sufficient to improve self‐esteem.

A potential confounder for findings was the integrated benefit of socialisation by life review. Some studies noted that life review group interventions assisted to satisfy unmet social needs for older adults in institutionalised care settings.^34^, ^91^, ^92^ There is a high prevalence of social isolation within older adults, with research estimating nearly one quarter of adults older than 65 are socially isolated^93^ and over 40% feel lonely.^94^ Given the substantial role loneliness and social isolation plays in decreased psychological well‐being within older adults,^95^ this is certainly an additional benefit of conducting life review in older adults. However, the question arises as to whether it is the supplementary socialisation that comes with life review that provides therapeutic benefit, as opposed to the actual content and concept of life review therapy. While life review can be helpful in stimulating socialisation within the elderly, it is perhaps less clear if any other intervention or social activity would have resulted in a comparable benefit or if it was life review therapy, in particular, that was responsible. Therefore, more research comparing life review with other psychotherapeutic and social activities would certainly help to ascertain and clarify the therapeutic benefits unique to life review therapies.

Due to the broad selection criteria dictated by the study design of this paper, all forms of life review were included regardless of format, style or variations in method. This allowed us to gain a broad and comprehensive review of all the existing literature, whereby consensus of the existing research could be synthesised, and future areas of research identified. This also allowed us to decrease the risk of selection bias. However, this broad selection criteria also had limitations which will be discussed in the limitations section.

### Future directions

It is clear therefore, that there are many areas where future research would be warranted. Very importantly, future research should focus more on sustained benefits, with more studies conducting longer follow up for participant groups. This is of particular importance, since the nature of life review therapy stipulates by necessity that it ends, unlike cognitive‐behavioural therapy, gestalt therapy and others, which are processes that incorporate the activities of every day as the foundations for continuing therapeutic work.^96^, ^97^ Life review therapy programs eventually conclude after all life stages are discussed^9^ and consequently may have little repeatability.

With growing life expectancies and growth of the elderly population, the number of people with dementia globally is also expected to substantially increase, with some studies estimating almost a tripling of the dementia population by 2050.^98^ This would be associated with a large economic burden globally, with an estimated annual growth rate 15.94% from 2000 to 2016.^99^ Compounded with the substantial costs to train therapists in psychotherapy techniques,^100^ it would therefore also be highly beneficial to research the feasibility and cost benefits of life review interventions. This is particularly important as life review would likely be used as a symptomatic or repeated therapy for this specific patient group.

It would also be beneficial to compare life review with existing psychotherapies to determine if it is superior to existing therapies. Complementarily, future studies should also compare life review interventions with other forms of socialisation to better understand the unique benefits of life review therapy itself. These are considerations which can potentially inform whether life review is economically and practically worthwhile implementing in nursing homes or other community environments. Finally, life review in older PTSD patients demonstrates promising results, and further research in this field would be valuable to confirm these findings and provide further evidence to support its use in this patient group.

### Limitations

During the review, we noted that life review therapy was known by many potential names including life history therapy, reminiscence therapy, and life story review. This was problematic for the soundness of the results since reminiscence therapy is a distinct modality and is separate from life review, although multiple studies conflated the two terms.^21^, ^23^, ^56^ While both life review and reminiscence therapy involve recollection and reminiscence, life review is chronologically structured and aims to also identify and integrate both positive and negative events to redirect repetitive rumination and reframe the participant's perspective on their life. Reminiscence therapy, on the other hand, uses select memories as a prompt to invoke positive emotion and stimulate socialisation, particularly in patients with dementia and cognitive decline.^101^ As a result of this, it is also possible there was missed literature during the search.

It also became apparent that the implementation of life review often differed in modality and format. While life review was commonly completed in individual environments, life review therapy varied greatly in duration in terms of number of sessions and duration of sessions themselves. There were also variations in number of participants, with life review conducted in individual, dyadic or group formats. Group therapies also varied in number of participants. While some studies noticed differences in efficacy depending on number of participants,^40^ there was little research comparing individual versus group versus dyadic therapy types. Additionally, therapies often varied in the mode of delivery, with the most common forms delivered orally. Other notable modes of delivery included written forms,^15^ video life review,^102^ computer supplemented,^16^ robot‐facilitated,^103^ caregiver‐administered,^76^ music‐based,^17^ collage‐based activities,^66^ storybook creation^58^ and theatre‐based^104^ methods. There were also variations in content, with life review therapies integrated with cognitive‐behavioural techniques,^39^, ^72^ spiritual/religious themes^75^, ^105^, ^106^, ^107^ or gestalt therapy techniques.^80^ Therapies were administered with varying levels of experience and while most studies utilised trained therapists to conduct therapy, others involved caregivers,^65^ home care workers,^71^ or family care givers,^76^ all with variable levels of experience, to administer therapy. All of these variations in the delivery of life review made studies difficult to compare and the observed therapeutic benefit more difficult to attribute to the content of life review alone.

## CONCLUSIONS

This study aimed to gain an understanding of the current existing literature for life review in older adults and uncover gaps in the current research. The search strategy produced 131 articles which were used to expose four themes: life satisfaction and self‐esteem, depression and depressive symptoms, institutional care and cognitive decline, and PTSD and trauma. Thematic analysis found that life review is being used successfully to improve depressive symptoms, life satisfaction and PTSD symptoms in the short‐term. There is evidence supporting that improvements in life satisfaction may be sustained. Life review has also been able to improve life satisfaction and depression for institutionalised elderly. Life review may have utility for persons with dementia over prolonged periods, but only for as long as the intervention is being administered. However, life review is unlikely to improve self‐esteem of participants. Future research should involve longer follow up to investigate whether benefits are sustained. Control groups in future studies should more often account for the supplementary socialisation from life review as a potential confounding factor. More research comparing life review therapy with other psychotherapy activities would also be beneficial. The current evidence for life review in PTSD patients is promising, therefore more research would be beneficial to substantiate and quantify these benefits.

## Funding

There is no funding associated with the work featured in this article. There were no grants associated with this research.

## Data Availability

Data sharing not applicable to this article as no datasets were generated or analysed during the current study.

## APPENDIX A SEARCH STRATEGY

**Table:** MeSH terms, keywords, databases, and search terms used

MeSH terms	Keywords
Life review; life history review Older adulthood; geriatric patients; aged	Life review; life review therapy; life history review Older adults; elderly; geriatric patients; aged; over 65; old age
Databases	Search terms
CINAHL, Medline, PsycINFO, Embase, Scopus	(life review* OR life review therap* OR life history review) AND (older adult* OR older person* OR elder* OR geriatric patients OR aged OR over 65)

## References

[1] United Nations Department of Economic and Social Affairs . World Social Report 2023. New York, NY: United Nations, 2023.

[2] Lowenthal MF . Social isolation and mental illness in old age. Am Sociol Rev 1964; 29: 54–70. 10.2307/2094641.

[3] Zenebe Y , Akele B , W/Selassie M , Necho M . Prevalence and determinants of depression among old age: a systematic review and meta‐analysis. Ann Gen Psychiatry 2021; 20: 55. 10.1186/s12991-021-00375-x. 34922595 PMC8684627

[4] Maier A , Riedel‐Heller SG , Pabst A , Luppa M . Risk factors and protective factors of depression in older people 65+. A systematic review. PLoS One 2021; 16: e0251326. 10.1371/journal.pone.0251326. 33983995 PMC8118343

[5] Roese N , Epstude K , Fessel F *et al*. Repetitive regret, depression, and anxiety: findings from a nationally representative survey. J Soc Clin Psychol 2009; 28: 671–688. 10.1521/jscp.2009.28.6.671.

[6] Butler R . The life review: an interpretation of reminiscence in the aged. Psychiatry 1963; 26: 65–76. 10.1080/00332747.1963.11023339. 14017386

[7] Erikson EH . Childhood and Society. New York, NY: W W Norton & Co, 1950; 397.

[8] Wong P , Watt L . What types of reminiscence are associated with successful aging. Psychol Aging 1991; 6: 272–279. 10.1037/0882-7974.6.2.272. 1863396

[9] Haight BK , Haight BS . The handbook of structured life review. Baltimore: Health Professions Press, 2007.

[10] Butler RN . Successful aging and the role of the life review. J Am Geriatr Soc 1974; 22: 529–535. 10.1111/j.1532-5415.1974.tb04823.x. 4420325

[11] Sherman E . Reminiscentia: cherished objects as memorabilia in late‐life reminiscence. Int J Aging Hum Dev 1991; 33: 89–100. 10.2190/fjw1-60uf-ww1r-fp2k. 1955210

[12] Haight BK , Michel Y , Hendrix S . The extended effects of the life review in nursing home residents. Int J Aging Hum Dev 2000; 50: 151–168. 10791613 10.2190/QU66-E8UV-NYMR-Y99E

[13] Ingersoll‐Dayton B , Kropf N , Campbell R , Parker M . A systematic review of dyadic approaches to reminiscence and life review among older adults. Aging Ment Health 2019; 23: 1074–1085. 10.1080/13607863.2018.1555696. 30596457

[14] Lewis MI , Butler RN . Life‐review therapy. Putting memories to work in individual and group psychotherapy. Geriatrics 1974; 29: 165–173. 4417455

[15] Chippendale T , Bear‐Lehman J . Effect of life review writing on depressive symptoms in older adults: a randomized controlled trial. Am J Occup Ther 2012; 66: 438–446. 10.5014/ajot.2012.004291. 22742692

[16] Preschl B , Maercker A , Wagner B *et al*. Life‐review therapy with computer supplements for depression in the elderly: a randomized controlled trial. Aging Ment Health 2012; 16: 964–974. 10.1080/13607863.2012.702726. 22788983

[17] Bennett SL , Maas F . The effect of music‐based life review on the life satisfaction and ego integrity of elderly people. Br J Occup Ther 1988; 51: 433–436.

[18] Butler RN . The life review: an unrecognized bonanza. Int J Aging Hum Dev 1981; 12: 35–38. 10.2190/wc4t-v05j-40g5-3m7e 7203669

[19] Whittemore R , Knafl K . The integrative review: updated methodology. Journal of Advanced Nursing 2005; 52: 546–553. 10.1111/j.1365-2648.2005.03621.x. 16268861

[20] Neugarten BL , Havighurst RJ , Tobin SS . The measurement of life satisfaction. J Gerontol 1961; 16: 134–143. 10.1093/geronj/16.2.134. 13728508

[21] Menn L , Corsten S , Lauer N , Wallace SJ . The effectiveness of biographical approaches in long‐term care: a systematic review. Gerontologist 2020; 60: e309–e328. 10.1093/geront/gnz074. 31175820

[22] Lan X , Xiao H , Chen Y . Effects of life review interventions on psychosocial outcomes among older adults: a systematic review and meta‐analysis. Geriatr Gerontol Int 2017; 17: 1344–1357. 10.1111/ggi.12947. 28124828

[23] Bohlmeijer E , Roemer M , Cuijpers P , Smit F . The effects of reminiscence on psychological well‐being in older adults: a meta‐analysis. Aging Ment Health 2007; 11: 291–300. 10.1080/13607860600963547. 17558580

[24] Gatz M , Fiske A , Fox LS *et al*. Empirically validated psychological treatments for older adults. J Ment Health 1998; 4: 9–46.

[25] Bharucha AJ , Dew MA , Miller MD , Borson S , Reynolds C III . Psychotherapy in long‐term care: a review. J Am Med Dir Assoc 2006; 7: 568–580. 10.1016/j.jamda.2006.08.003. 17095422

[26] Capps HE . A comparison of the effects of life review and reminiscence group counseling on depression, life satisfaction and self‐esteem of older persons. Dissertation Abstracts International Section A: Humanities and Social Sciences 1998; 59: 733.

[27] Haight BK . The therapeutic role of a structured life review process in homebound elderly subjects. J Gerontol 1988; 43: P40–P44. 2964468 10.1093/geronj/43.2.p40

[28] Gonçalves DC , Albuquerque PB , Paul C . Life review with older women: an intervention to reduce depression and improve autobiographical memory. Aging Clinical Exp Res 2009; 21: 369–371. 10.1007/bf03324931. 19959930

[29] Chiang K , Lu R , Chu H , Chang Y , Chou K . Evaluation of the effect of a life review group program on self‐esteem and life satisfaction in the elderly. Int J Geriatr Psychiatry 2008; 23: 7–10. 10.1002/gps.1824. 17477451

[30] Xiuyan L , Huimin X , Ying C , Xiaoling Z . Effects of life review intervention on life satisfaction and personal meaning among older adults with frailty. J Psychosoc Nurs Ment Health Serv 2018; 56: 30–36. 10.3928/02793695-20180305-01. 29538790

[31] Subramaniam P , Woods B , Whitaker C . Life review and life story books for people with mild to moderate dementia: a randomised controlled trial. Aging Ment Health 2014; 18: 363–375. 10.1080/13607863.2013.837144. 24063317 PMC4017276

[32] Sharif F , Jahanbin I , Amirsadat A , Moghadam MH . Effectiveness of life review therapy on quality of life in the late life at day care centers of shiraz, Iran: a randomized controlled trial. International Journal of Community Based Nursing & Midwifery 2018; 6: 136–145. 29607342 PMC5845117

[33] Arland WT . A life review with elderly subjects assessing for impact on depression and life satisfaction. Dissertation Abstracts International Section A: Humanities and Social Sciences 1991; 51: 2485.

[34] Chippendale T . The effects of life review through writing on depressive symptoms and life satisfaction in older adults. Dissertation Abstracts International: Section B: The Sciences and Engineering 2012; 73: 237.

[35] Haber D . Life review: implementation, theory, research, and therapy. Int J Aging Hum Dev 2006; 63: 153–171. 10.2190/da9g-rhk5-n9jp-t6cc. 17137032

[36] Jeffers SL , Hill R , Krumholz MF , Winston‐Proctor C . Themes of gerotranscendence in narrative identity within structured life review. GeroPsych 2020; 33: 77–84. 10.1024/1662-9647/a000235.

[37] Wacks VQ . Guided autobiography with the elderly. J Appl Gerontol 1989; 8: 512–523. 10.1177/073346488900800408.

[38] Jordan CH . Rosenberg Self‐Esteem Scale. In: Zeigler‐Hill V , Shackelford TK , eds. Encyclopedia of Personality and Individual Differences. Cham, Switzerland: Springer International Publishing, 2020; 4518–4520.

[39] Schwartz‐Oscar SJ . Comparison of a cognitive behavioral plus life review intervention and a life review only intervention for retired persons over age 65. Dissertation Abstracts International: Section B: The Sciences and Engineering 2013; 74: 1–104.

[40] Silver MH . Life review as a developmental process: themes of caring, mourning, and integrity in group and individual therapy with low‐income elderly women. Diss Abstr Int 1983; 43: 3743.

[41] Lee Y , Tabourne CES , Yoon J . Effects of life review program on emotional well‐being of Korean elderly with Alzheimer's disease. Am J Recreation Ther 2008; 7: 35–45.

[42] Malde S . Guided autobiography: a counseling tool for older adults. J Couns Dev 1988; 66: 290–293. 10.1002/j.1556-6676.1988.tb00872.x.

[43] Stevens‐Ratchford RG . The effect of life review reminiscence activities on depression and self‐esteem in older adults. Am J Occup Ther 1993; 47: 413–420. 8498465 10.5014/ajot.47.5.413

[44] Tabourne CE . The effects of a life review program on disorientation, social interaction and self‐esteem of nursing home residents. Int J Aging Hum Dev 1995; 41: 251–266. 8666469 10.2190/EG53-878E-MGRK-BCPP

[45] Haight BK , Michel Y , Hendrix S . Life review: preventing despair in newly relocated nursing home residents short‐ and long‐term effects. Int J Aging Hum Dev 1998; 47: 119–142. 10.2190/A011-BRXD-HAFV-5NJ6. 9836092

[46] Miller HM . Life review as an intervention: a comparison of a systematically induced life review and non‐specific factors groups with elderly adults. Diss Abstr Int 1986; 47: 381–382.

[47] Lan X , Xiao H , Chen Y . Life review for Chinese older adults in nursing homes: cultural acceptance and its effects. Int Psychogeriatr 2019; 31: 527–535. 10.1017/S1041610218001084. 30277193

[48] Westcott NA . Application of the structured life‐review technique in counseling elders. Personnel & Guidance J 1983; 62: 180–181. 10.1111/j.2164-4918.1983.tb00181.x.

[49] Tabourne CES . The life review program as an intervention for an older adult newly admitted to a nursing home facility: a case study. Ther Recreation J 1995; 29: 228–236.

[50] Silver MH . The significance of life review in old age. J Geriatr Psychiatry 2002; 35: 11–23.

[51] Stevens GL , Kaas MJ , Hjartardottir KL . Psychotherapy with older adults. In: Psychotherapy for the Advanced Practice Psychiatric Nurse: A how‐to Guide for Evidence‐Based Practice, 3rd edn. New York, NY: Springer Publishing Company, 2022; 823–865.

[52] Holvast F , Massoudi B , Oude Voshaar RC , Verhaak PFM . Non‐pharmacological treatment for depressed older patients in primary care: a systematic review and meta‐analysis. PLoS One 2017; 12: e0184666. 10.1371/journal.pone.0184666. 28938015 PMC5609744

[53] Al‐Ghafri BR , Al‐Mahrezi A , Chan MF . Effectiveness of life review on depression among elderly: a systematic review and meta‐analysis. *The* Pan African Medical Journal 2021; 40: 168. 10.11604/pamj.2021.40.168.30040. 34970410 PMC8683455

[54] Baba H , Kito S , Nukariya K *et al*. Guidelines for diagnosis and treatment of depression in older adults: a report from the Japanese society of mood disorders. Psychiatry Clin Neurosci 2022; 76: 222–234. 10.1111/pcn.13349. 35274788

[55] Bohlmeijer E , Smit F , Cuijpers P . Effects of reminiscence and life review on late‐life depression: a meta‐analysis. Int J Geriatr Psychiatry 2003; 18: 1088–1094. 10.1002/gps.1018. 14677140

[56] Zuiderveen A , Ivey C , Dordan S , Leiras C . Encouraging occupation: a systematic review of the use of life review and reminiscence therapy for the treatment of depressive symptoms in older adults. Occupational Therapy Mental Health 2016; 32: 281–298. 10.1080/0164212X.2016.1145090.

[57] Chen Y‐j , Li X‐x , Pan B *et al*. Non‐pharmacological interventions for older adults with depressive symptoms: a network meta‐analysis of 35 randomized controlled trials. Aging Ment Health 2021; 25: 773–786. 10.1080/13607863.2019.1704219. 31880174

[58] Ng SE , Tien A , Thayala JNV , Ho RCM , Chan MF . The effect of life story review on depression of older community‐dwelling Chinese adults in Singapore: a preliminary result. Int J Geriatr Psychiatry 2013; 28: 328–330. 10.1002/gps.3851. 23382101

[59] Mastel‐Smith BA , McFarlane J , Sierpina M , Malecha A , Haile B . Improving depressive symptoms in community‐dwelling older adults: a psychosocial intervention using life review and writing. J Gerontol Nurs 2007; 33: 13–19. 10.3928/00989134-20070501-04. 17511331

[60] Forstmeier S , Zimmermann S , van der Hal E *et al*. Effect of life review therapy for holocaust survivors: a randomized controlled trial. J Trauma Stress 2023; b1b: 628–641. 10.1002/jts.22933. 37155933

[61] Hanaoka H , Okamura H . Study on effects of life review activities on the quality of life of the elderly: a randomized controlled trial. Psychother Psychosom 2004; 73: 302–311. 10.1159/000078847. 15292628

[62] Bazrafshan M‐R , Faramarzian Z , Jokar M *et al*. The effect of reminiscence on depression in elderly people with suicidal ideation: a randomized controlled trial. Jundishapur J Chronic Disease Care 2022; 11: 1–9. 10.5812/jjcdc-130420.

[63] Serrano Selva JP , Latorre Postigo JM , Ros Segura L *et al*. Life review therapy using autobiographical retrieval practice for older adults with clinical depression. Psicothema 2012; 24: 224–229. 22420349

[64] Daniels LR , Boehnlein J , McCallion P . Aging, depression, and wisdom: a pilot study of life‐review intervention and PTSD treatment with two groups of Vietnam veterans. J Gerontol Soc Work 2015; 58: 420–436. 10.1080/01634372.2015.1013657. 25751708

[65] Miyawaki CE , Tahija N , McClellan A , Chen N‐W . Feasibility study of caregiver‐provided life review: implementation, adaptation, and effects on care Recipients' depressive symptoms. Clin Gerontol 2022: 1–12. 10.1080/07317115.2022.2144578. 36369922

[66] Sakaguchi S , Okamura H . Effectiveness of collage activity based on a life review in elderly cancer patients: a preliminary study. Palliat Support Care 2015; 13: 285–293. 10.1017/S1478951514000194. 24762444

[67] Matteson MA , Munsat EM . Group reminiscing therapy with elderly clients. Issues Ment Health Nurs 1982; 4: 177–189. 6926412 10.3109/01612848209141054

[68] Moon Fai C , P. Leong KS , Boon Ling H *et al*. Reducing depression among community‐dwelling older adults using life‐story review: a pilot study. Geriatr Nurs 2014; 35: 105–110. 10.1016/j.gerinurse.2013.10.011. 24246689

[69] Watt LM , Cappeliez P . Integrative and instrumental reminiscence therapies for depression in older adults: intervention strategies and treatment effectiveness. Aging Ment Health 2000; 4: 166–177. 10.1080/13607860050008691.

[70] Blankenship LM , Molinari V , Kunik M . The effect of a life review group on the reminiscence functions of geropsychiatric inpatients. Clin Gerontol 1996; 16: 3–18. 10.1300/j018v16n04_02.

[71] Mastel‐Smith B , Binder B , Malecha A , Hersch G , Symes L , McFarlane J . Testing therapeutic life review offered by home care workers to decrease depression among home‐dwelling older women. Issues Ment Health Nurs 2006; 27: 1037–1049. 10.1080/01612840600943689. 17050337

[72] Puentes WJ . Cognitive therapy integrated with life review techniques: an eclectic treatment approach for affective symptoms in older adults. Journal of Clinical Nursing (Wiley‐Blackwell) 2004; 13: 84–89. 10.1046/j.1365-2702.2003.00828.x. 14687297

[73] Luo Y , Sun F , Jiang L , Zhang A . The stress and coping experiences among Chinese rural older adults in welfare housing: through the lens of life review. Aging Ment Health 2019; 23: 1086–1094. 10.1080/13607863.2019.1612322. 31079480

[74] Hendriks L , Veerbeek MA , Volker D , Veenendaal L , Willemse BM . Life review therapy for older adults with depressive symptoms in general practice: results of a pilot evaluation. Int Psychogeriatr 2019; 31: 1801–1808. 10.1017/S1041610219000218. 31032754

[75] Richards‐Campbell JM . The effects of a life review reminiscence/redemption curriculum on depression and spiritual well‐being in the elderly. Dissertation Abstracts International Section A: Humanities and Social Sciences 2004; 65: 1067.

[76] Miyawaki CE , Brohard C , Chen N‐W , Rubin A , Willoughby S . Can family caregivers provide life review to reduce depression in older adults with dementia? J Gerontol Nurs 2020; 46: 41–48. 10.3928/00989134-20200108-04. 31978238

[77] Masten‐McGilvray VL . The effect of group life review therapy on adaptation in the elderly: a comparison of the relative efficacy of life review and reminiscence. Diss Abstr Int 1990; 51: 3139.

[78] Fortier‐Buckley L . The effects of a short‐term Eriksonian group life review on ego integrity versus despair in the elderly. Dissertation Abstracts International: Section B: The Sciences and Engineering 1994; 55: 589.

[79] Fagerstrom K . The differential effects of guided autobiography types on well‐being in the elderly. Dissertation Abstracts International: Section B: The Sciences and Engineering 2013; 73: 1–83.

[80] Koffman SD . Structured reminiscence and gestalt life review: group treatment of older adults for late life adjustment. Dissertation Abstracts International Section A: Humanities and Social Sciences 1998; 59: 737.

[81] Kiernat JM . The use of life review activity with confused nursing home residents. Am J Occup Ther 1979; 33: 306–310. 474339

[82] Haight BK . The Structured Life‐Review Process: A Community Approach to the Aging Client. In: Care Giving in Dementia. London: Routledge, 2014: 272–292.

[83] Zimmermann S , van der Hal E , Auerbach M *et al*. Life review therapy for holocaust survivors: two systematic case studies. Psychotherapy 2021; 59: 521–532. 10.1037/pst0000419. 34941339

[84] McInnis‐Dittrich K . Adapting life‐review therapy for elderly female survivors of childhood sexual abuse. Families in Society 1996; 77: 166–173. 10.1606/1044-3894.891.

[85] Winterstein TB , Avieli H , Gichaz M . Recovering the lost soul: older Women's reflections on past Intrafamilial child sexual abuse. Qual Health Res 2023; 33: 426–439. 10.1177/10497323231159802. 36882288

[86] Pelts MD , Hrostowski S , Cardin SA , Swindle R . Using a life review to inform mental health services with older lesbian and gay veterans. Best Practices Mental Health: An Int J 2018; 14: 27–39.

[87] Maercker A , Bachem R . Life‐review interventions as psychotherapeutic techniques in psychotraumatology. Eur J Psychotraumatol 2013; 4: 1–9. 10.3402/ejpt.v4i0.19720. PMC3660622 23700490

[88] Maercker A . Life‐review technique in the treatment of PTSD in elderly patients: rationale and three single case studies. J Clin Geropsychol 2002; 8: 239–249. 10.1023/A:1015952429199.

[89] Westwood MJ , McLean HB . Traumatic memories and life review. In: Transformational reminiscence: Life story work. New York: Springer Publishing Company, 2007.

[90] Sable LM . Life review therapy: an occupational therapy treatment technique with geriatric clients. Physical Occupational Therapy Geriatrics 1985; 3: 49–54.

[91] Chan KY , Lau VWK , Cheung KC , Chang RSK , Chan ML . Reduction of psycho‐spiritual distress of an elderly with advanced congestive heart failure by life review interview in a palliative care day center. SAGE Open Med Case Rep 2016; 4: 4. 10.1177/2050313X16665998. PMC5006298 27621805

[92] Dahley L , Sanders GF . Use of a structured life review and its impact on family interactions. Activities, Adaptation & Aging 2016; 40: 53–66. 10.1080/01924788.2016.1127060.

[93] National Academies of Sciences E, Medicine . Social isolation and loneliness in older adults: opportunities for the health care system. Washington, DC: National Academies Press, 2020: 316. 32510896

[94] Perissinotto CM , Stijacic Cenzer I , Covinsky KE . Loneliness in older persons: a predictor of functional decline and death. Arch Intern Med 2012; 172: 1078–1083. 10.1001/archinternmed.2012.1993. 22710744 PMC4383762

[95] Alpass FM , Neville S . Loneliness, health and depression in older males. Aging Ment Health 2003; 7: 212–216. 10.1080/1360786031000101193. 12775403

[96] Craske MG . Cognitive–behavioral therapy (2nd ed.). Washington, DC: American Psychological Association, 2017.

[97] Perls F , Hefferline G , Goodman P . Gestalt Therapy. In: Nelson‐Jones R , ed. Six key approaches to counselling and Therapy. Dell, New York: Continuum, 1951; 19–313.

[98] GBD 2019 Dementia Forecasting Collaborators . Estimation of the global prevalence of dementia in 2019 and forecasted prevalence in 2050: an analysis for the global burden of disease study 2019. Lancet Public Health 2022; 7: e105–e125. 10.1016/s2468-2667(21)00249-8. 34998485 PMC8810394

[99] Xu J , Zhang Y , Qiu C , Cheng F . Global and regional economic costs of dementia: a systematic review. Lancet 2017; 390: S47. 10.1016/S0140-6736(17)33185-9.

[100] Okamura KH , Benjamin Wolk CL , Kang‐Yi CD *et al*. The Price per prospective consumer of providing therapist training and consultation in seven evidence‐based treatments within a large public behavioral health system: an example cost‐analysis metric. Front Public Health 2017; 5: 356. 10.3389/fpubh.2017.00356. 29359126 PMC5766669

[101] Woods B , O'Philbin L , Farrell EM , Spector AE , Orrell M . Reminiscence therapy for dementia. Cochrane Database Syst Rev. 2018; 3: Cd001120. 10.1002/14651858.CD001120.pub3. 29493789 PMC6494367

[102] Hargrave TD . Using video life reviews with older adults. J Fam Ther 1994; 16: 259–268. 10.1111/j.1467-6427.1994.00794.x.

[103] Ueda A , Hideyuki T , Yuichiro Y , Hiroshi I , Haruo N . Do robots facilitate life review narratives of older adults? A preliminary study. Gerontechnology 2021; 20: 1–12. 10.4017/gt.2021.20.2.28-470.09.34305492

[104] Perlstein S . Transformation: life review and communal theater. J Gerontol Soc Work 1988; 12: 137–148. 10.1300/J083v12n03_10.

[105] Stinson AM . Spiritual life review with older adults: finding meaning in late life development. Dissertation Abstracts International Section A: Humanities and Social Sciences 2014; 74: 1–171.

[106] Fountain DE . Life review groups: an experimental study of religiosity, life satisfaction and concomitant behavioral changes in residents of long‐term care facilities. Dissertation Abstracts International Section A: Humanities and Social Sciences 1993; 53: 3674.

[107] Baker DC . The investigation of pastoral care interventions as a treatment for depression among continuing care retirement community residents. J Relig Gerontol 2001; 12: 63–85. 10.1300/J078v12n01_06.

